# Cholesterol Transport Dysfunction and Its Involvement in Atherogenesis

**DOI:** 10.3390/ijms23031332

**Published:** 2022-01-25

**Authors:** Anastasia V. Poznyak, Dmitry A. Kashirskikh, Vasily N. Sukhorukov, Vladislav Kalmykov, Andrey V. Omelchenko, Alexander N. Orekhov

**Affiliations:** 1 Institute for Atherosclerosis Research, Osennyaya Street 4-1-207, 121609 Moscow, Russia; omi@bk.ru; 2 Laboratory of Angiopathology, Institute of General Pathology and Pathophysiology, Russian Academy of Medical Sciences, 125315 Moscow, Russia; dim.kashirsckih@gmail.com (D.A.K.); xxor2011@gmail.com (V.K.); 3 AP Avtsyn Research Institute of Human Morphology, 3 Tsyurupa Street, 117418 Moscow, Russia; vnsukhorukov@gmail.com

**Keywords:** atherosclerosis, cholesterol, cholesterol transport, LDL

## Abstract

Atherosclerosis is the cause of the development of serious cardiovascular disorders, leading to disability and death. Numerous processes are involved in the pathogenesis of atherosclerosis, including inflammation, endothelial dysfunction, oxidative stress, and lipid metabolism disorders. Reverse transport of cholesterol is a mechanism presumably underlying the atheroprotective effect of high-density lipoprotein. In this review, we examined disorders of cholesterol metabolism and their possible effect on atherogenesis. We paid special attention to the reverse transport of cholesterol. Transformed cholesterol metabolism results in dyslipidemia and early atherosclerosis. Reverse cholesterol transport is an endogenous mechanism by which cells export cholesterol and maintain homeostasis. It is known that one of the main factors leading to the formation of atherosclerotic plaques on the walls of blood vessels are multiple modifications of low-density lipoprotein, and the formation of foam cells following them.

## 1. Cholesterol and Lipoprotein Metabolism

The most vital lipid element of almost all cell membranes is cholesterol. It is also a predecessor of diverse steroid hormones, such as sex hormones (estrogen, testosterone, and progesterone) and corticosteroids (corticosterone, cortisol, cortisone, and aldosterone) [1]. Moreover, during the biosynthesis of bile acids, cholesterol is mainly turning into bile acids. Along with the simultaneous secretion of cholesterol and bile acids into bile, this stage results in a reduction of cholesterol deposition in the plasma and helps to remove excess cholesterol from the body [2].

Due to the fact that almost all cells of the body require an uninterrupted input of cholesterol, a complex series of transport, biosynthetic, and regulatory mechanisms has developed in human beings. Usually, cholesterol is obtained as a result of dietary and bile cholesterol uptake, which occurs in the intestine, and synthesized de novo from acetyl-CoA in the body [2,3]. In addition, cholesterol is normally obtained by absorption of dietary and bile cholesterol in the intestine and newly synthesized de novo from acetyl CO_2_ in the body. If there is a shortage of CO_2_ and water in the human body, cholesterol cannot be metabolized, since there are no enzymes in human tissues that can destroy the ring structure. Therefore, in order to avoid the potentially dangerous cholesterol storage in the human body, the abundance of cholesterol must be metabolized into other compounds and/or excreted in the feces [4]. Elimination of cholesterol from cells could be achieved by high-density lipoprotein (HDL) contacting with the plasmatic membrane or by the formation of very low-density lipoprotein (VVLDL), which is secreted through the secretory pathway.

This truly difficult problem is often solved by modifying certain groups of substituents in the hydrocarbon tail or in the ring structure of the cholesterol molecule. Thus, most of the cholesterol is excreted from the human’s body in the form of unchanged molecules (i.e., both in non-esterified and esterified forms) or after biochemical modification with other steroid products (e.g., bile acids and steroid hormones) [5]. Several pathways were found for the net flow of cholesterol through the main human tissue departments, which reflects that in an adult, the cholesterol pool is maintained in an almost uninterrupted manner. There are two pathways in which new cholesterol is able to join an already existing cholesterol pool: (1) cholesterol taken up from food and bile sources through epithelial cells of the small intestine, (2) and newly synthesized cholesterol in various body tissues [6]. The majority of cholesterol is synthesized by the liver and brain, but it is still not completely clear how the brain eliminates excess cholesterol.

The availability of dietary and bile cholesterol to the body (including the same species) is quite different between various animal species, and the amount of dietary cholesterol consumed also significantly varies over a day [7]. The aggregate amount of cholesterol from the small intestine usually depends on the absorption efficiency of intestinal cholesterol and the amount of cholesterol consumed each day. In addition, bile cholesterol is reabsorbed by the small intestine, which, in turn, gives about two-thirds of the aggregate amount of cholesterol coming from the intestine daily. In different animals (including animals of the same species), the rate of cholesterol biosynthesis in the liver is very distinct [8]. Cholesterol uptake from the small intestine is able to control the synthesis of liver cholesterol depending on the amount of daily food intake. In addition, the cholesterol molecule can be metabolized into other compounds, for example, bile acids, which are excreted from the body through the intestinal tract and ultimately converted into stool [9]. It is important to pay attention to the fact that the sterol outflow of transporters ABCG5 and ABCG8 on the canalicular membrane of hepatocytes is responsible for the regulation of hepatic secretion of bile cholesterol, and bile acid export pumps—ABCB11—are responsible for hepatic secretion of bile acids. These transporters in the liver are of key importance in regulating the excretion of excess cholesterol from the body, which is able to be excreted in the form of non-esterified cholesterol or in the form of its metabolic products: bile acids [10]. Interestingly, in children and growing animals, the intake of cholesterol into the body far exceeds its output. This is due to the fact that a net accumulation of cholesterol is required to maintain an elevated body weight. With the onset of adulthood, it is vital to maintain a balance between the intake and output of cholesterol; that is, both the intake and output of cholesterol should be equal [7].

In summary, in order to regulate the rate of cholesterol biosynthesis in the body and the rate of cholesterol excretion, the regulatory mechanisms of cholesterol metabolism should remain functional, being able to adapt to different amounts of cholesterol that are absorbed by the small intestine at different times [11]. As a rule, these regulatory mechanisms of cholesterol metabolism function perfectly. Thus, excess cholesterol does not build up in the body, but there is always enough cholesterol in stock to fulfill the metabolic needs of different cells. Nevertheless, even minor imbalances result in higher concentrations of cholesterol in plasma and/or hypersecretion of cholesterol in the liver in humans [12].

In the cardiovascular system, such a metabolic anomaly usually causes the accumulation of excess cholesterol ester molecules in the arterial wall, which results in clinically obvious atherosclerosis, affecting mainly the heart and brain arteries [13]. It is important to note that cholesterol does not accumulate in muscular-type arteries or elastic-type lung arteries.

Lipoproteins in plasma include a heterogeneous mixture of particles that can be differentiated by size, density, and buoyancy. In the 1950s, analytical ultracentrifugation was applied for the first time, aiming to separate lipoprotein particles according to their migration rate in an intense centrifugal field, i.e., HDL_2_(F_1–20_ 3.5–9.0), HDL_3_ (F_1.20_ 0–3.5), LDL (S_f_ 0–12), intermediate-density lipoproteins (IDLs, S_f_ 12–20), small VLDL (S_f_ 20–100), and large VLDL (S_f_ 100–400) [14]. So far, a similar approach has been used to measure these lipoprotein particles. For example, the same approach was widely used in the 2010s. Analytical ultracentrifugation is the canonical model by which other methods are calibrated. Despite the fact that the instruments used for analyzing the mass concentration of plasma lipoproteins within individual flotation intervals have been further improved, the basic methodology and the aforementioned density intervals remain unchanged when they are used for basic and clinical studies [15].

In the 1950s and 1960s, a prospective study of male employees at the Livermore Radiation Laboratory was conducted. Over 10 years of follow-up, it was demonstrated that in contrast to the average plasma amount of the total sample in 38 men who developed coronary heart disease (CHD), the HDL level was 32% lower than HDL2 (*p* < 0.01), 8% lower than HDL3 (*p* = 0.02), 13% higher than LDL (*p* < 0.001), 23% higher than LDL (*p* < 0.001), and 21% higher than small LDL (*p* < 0.01) [16]. In men with CHD, the mass concentration of VLDL in blood plasma was 14% higher compared to the main population. This prospective study was the first study to link HDL subfractions with coronary heart disease development. It was also identified that the relationship between coronary heart disease and total cholesterol, HDL, and LDL decreases with aging, and especially after 50 years [17].

Prospective population studies have also demonstrated that for patients with coronary heart disease and stroke, the concentration of LDL cholesterol in blood plasma is a good sign. In addition, studies on interventions with statins have proved that they lead to a decrease in LDL cholesterol and risk of coronary heart disease and stroke development. It is worth noting that no aggressive statin therapy is able to cause a reduction in CHD or stroke risk [18]. Subsequent prospective population studies have also revealed that for patients with CHD and stroke, low HDL cholesterol acts as a standalone predictor. This relationship remains even under for therapeutic interventions with statins in which the level of LDL cholesterol reaches a maximum low level [19]. Despite the fact that HDL in relation to the development of atherosclerosis demonstrates several promising atheroprotective effects, at the moment, no direct evidence has been obtained through human clinical trials indicating that an elevation in the HDL cholesterol concentration results in a reduction in the prevalence of CHD and stroke [20].

At the same time, animal experiments and human studies have proved that therapy that increases HDL levels is able to reduce the progression or even contribute to the regression of atheroma. These studies have launched an extensive research effort to identify therapeutic interventions that are able to elevate the concentration of HDL cholesterol in plasma as effectively as statins reduce the concentration of LDL cholesterol in plasma [21].

## 2. Reverse Cholesterol Transport

Even though it is accepted that HDL performs many functions, its ability to stimulate reverse cholesterol transport is often considered essential for its atheroprotection. This was the motivation for conducting various studies on improving reverse cholesterol transport [22]. Even taking into account that this pathway has been actively studied over the past few years, the mechanistic understanding of lipid export mediated by the ABC family and nascent HDL biogenesis is still imperfect, and key pieces of the puzzle, such as the structural information of the “ABC subfamily”, are only arising [23].

ABCA1 mainly lipidizes small HDL particles, in particular ApoA-I, with the formation of incipient HDL, and ABCG1 stimulates the net outflow of cholesterol into larger HDL but not into lipid-poor ApoA-I80. In addition, ABCA1 trafficking between the cell surface and late endocytic vesicles has a function that is important for stimulating the outflow of cholesterol from endosomal/lysosomal compartments to lipid-free ApoA-I while ABCG1 is an intracellular sterol transporter that helps transfer cholesterol from the endoplasmic reticulum to the plasma membrane [24]. The outflow to HDL includes inactive diffusion of cholesterol, active transfer of cholesterol, and ABCA1 and ABCG1, and unbound receptors-absorbers of class B type 1 (SR-B1) mediate the transfer of lipids to HDL [25].

The next step after the transfer of cholesterol into HDL particles in HDL biology is the esterification of the acquired cholesterol with lecithin: cholesterol acyltransferase (LCAT) with the formation of cholesteryl ester, leading to mature HDL. Modernization of HDL particles is able to happen by hydrolysis of HDL triglycerides and phospholipids mediated by hepatic lipase and endothelial lipase [26]. In humans (but not in mice), cholesteryl ester in the HDL nucleus is able to migrate to triglyceride-saturated lipoproteins with the help of the cholesteryl ester transfer protein (CETP) for excretion through hepatic clearance in the liver through LDL or selective absorption through SR-B1, acting as a hepatic receptor for cholesteryl ester on HDL. Thus, the introduction of cholesterol into the liver, obtained from peripheral cells in humans, has two pathways: (1) direct (HDL-SR-B1) and (2) indirect (HDL-LDL/VLDL-LDLR liver). In the liver, cholesteryl ester is hydrolyzed, and free cholesterol is either turned into bile acids or transported by ABCG5 and ABCG8 into bile for excretion with feces [27].

Three conceptual approaches to enhance reverse cholesterol transport were presented: (1) to improve the outflow of cholesterol from macrophages, (2) to improve the functionality of HDL (i.e., its ability to receive or transport cholesterol); (3) and to improve the absorption of cholesterol in the liver and excretion of bile/intestines [28]. During the course of the research, the third approach was proved by growing evidence that several HDL-independent pathways are able to contribute to reverse cholesterol transport and that cholesterol elimination from the body does not require cholesterol elimination from the hepatobiliary system. Therefore, nowadays, reverse cholesterol transport covers all possible ways that pure cholesterol can flow from peripheral tissues to feces, including artificial ones with therapeutic potential. For instance, non-HDL particles consisting of 2-hydroxypropyl-β-cyclodextrin (CD) are artificial cholesterol acceptors and mediate reverse cholesterol transport and protection against atherosclerosis [29].

Alternative pathways that elevate reverse cholesterol transport involve liposomes: erythrocyte compartment, which is able to operate as a cholesterol absorber to elevate reverse cholesterol transport, cholesterol output mediated by microparticles, and synthetic nanoparticles and HDL mimetics, which not only serve to package and deliver therapeutic drugs (e.g., LXR agonists or statins) to the arterial wall to stimulate cholesterol outflow, but can also extract cholesterol plaques [30]. Efforts are also being made to elevate the activity of LCAT so that more free cholesterol can be esterified by elevating the number loaded into HDL [31].

A study demonstrated that SRB1 mutations in humans are related to a high risk of CD, despite elevated levels of HDL-C. This corresponded to mice in which SR-B1 deficiency, on the one hand, elevated HDL-C levels, and on the other hand, paradoxically increased atherosclerosis [32]. In these studies, SR-B1 removal or loss of function disrupted reverse cholesterol transport, which is consistent with a growing body of evidence indicating that, unlike absolute HDL-C concentrations, HDL function and cholesterol flow are the best factors determining atheroprotection. Nevertheless, it is worth paying attention to the fact that another study demonstrated that infrequent mutations disrupting the function of SR-B1 are associated not with the risk of CAD developing but with HDL-C [22].

Apart from the hepatobiliary cholesterol excretion pathway, there is a transintestinal cholesterol efflux (TICE). While the release of hepatobiliary cholesterol involves the transfer of cholesterol from hepatocytes to the bile ducts, TICE cholesterol migrates from the blood through enterocytes into the intestinal lumen [33]. It is estimated that these fecal cholesterol pathways—hepatobiliary and TICE—account for 65% and 35% of cholesterol removal in humans. Important regulators of cholesterol release are the receptors of the nuclear hormones LXR and the farnesoid X receptor (FXR), which control the transcription and activity of a significant number of cholesterol transporters and bile synthesis enzymes [34].

Although cholesterol can be independently excreted into bile for elimination from the body, in mammals, the main pathway for cholesterol catabolism is the synthesis and elimination of bile acids. Therefore, LXR and FXR are potential therapeutic targets for stimulating the secretion of TICE and biliary cholesterol and promoting reverse cholesterol transport [35]. Since the activation of LXR in the liver also triggers lipogenesis, which results in steatohepatitis, the development of a strategy for selective activation of nuclear receptors in the intestinal lumen to stimulate TICE without inducing lipogenesis in the liver represents a targeted approach to circumvent this problem. Moreover, miRNAs add an additional level of regulation of cholesterol metabolism by exercising post-transcriptional adverse control over certain genes, including ABCB11 and ATP8B1, suggesting anti-miRNA therapies [36].

## 3. Cholesterol in Atherosclerosis

Atherosclerosis is a complex pathology originating from an interplay of inflammation, lipid metabolism alterations, endothelial dysfunction, oxidative stress, and other various mechanisms. This disease is characterized by progressive artery narrowing, which leads to different adverse effects, from cardiovascular disease to acute myocardial infarction.

Transformed cholesterol metabolism results in dyslipidemia and early atherosclerosis, a process modulated by innate and adaptive immunity. It is worth noting that the formation of atherosclerotic lesions is preceded by the storage of apolipoprotein-containing lipoproteins, mainly LDL, in the subendothelial space [37]. These lipoproteins are subject to modification [38] by oxidation [39], acetylation [40], and aggregation [41], which leads to strengthening of their proinflammatory effect. Moreover, the decrease in the sialic acid component is an important contributor to atherogeneity. In apoprotein B-100 (apoB-100) of desialylated LDL, the amino group domain is chemically modified, whereas another domain is masked due to the changes in the tertiary structure of apoB-100 [39,42]. However, endothelial dysfunction is a key factor that determines the retention of lipoproteins, which is especially important in areas of the vascular bed with high shear stress, and in some conditions (for example, aging, low-severity inflammation, or oxidative stress caused by other pathological conditions, such as hypertension or diabetes) [43,44,45]. Disturbed shear stress has an impact on the site selectivity of atherosclerotic plaque formation because of the association with vessel wall remodeling affecting plaque vulnerability, stent restenosis, and smooth muscle cell intimal hyperplasia in venous bypass grafts [46]. Initially, lipoprotein accumulation adversely affects endothelial dysfunction, which is featured by the expression of adhesion molecules and the permeability of the endothelial layer. Thus, there is an increase in the adhesion, retention, and migration of immune cells in the subendothelial space [47].

Preserved monocytes differentiate into macrophages, obtaining either an anti-inflammatory phenotype, which favors a more sustainable and fibrous plaque, or a proinflammatory phenotype, outpacing ApoB-containing lipoproteins and transforming into foam cells, which in turn form fatty streak when accumulated [48]. At this phase, macrophages are modulated by modified LDL. In total, activation of the inflammasome (NLRP3); elevated release of cytokines, such as interleukin (IL)1β, IL6, and tumor necrosis factor (TNF)α; and the production of active forms of oxidants (ROS) contributes to the maintenance of atheroma inflammation [49]. However, ABCA1- and ABCG1-mediated cholesterol outflow pathways to lipid-poor HDL activated by steroid-regulated LXR transcription factors in macrophages improve atheroma formation and suppress NLRP3 activation [50].

Once the lesions reach the middle phase of development, they attack VSMCs (vascular smooth muscle cells), which differentiate into various phenotypes, such as macrophage-like cells saturated with lipids, with a proinflammatory, migrating, and proliferative phenotype [51]. VSMCs can also receive myofibroblasts features, which, along with the extracellular matrix, form a fibrous cap over the lipid nucleus, consisting of lipid-loaded macrophages. Monocytes are also able to differentiate into dendritic cells (DC), which favor cytokine signaling, inhibition of T cells (Treg), and regulatory and antigen-presenting stimulation of effector T helper (Th) cells [52].

As the lesion develops, the formation of obstructive unprotected plaques depends on the balance of different subsets of T cells. Th1 and Th17 cells are proinflammatory and proatherogenic; Th2 cells release anti-inflammatory cytokines; Treg cells inhibit the activity of CD4+Th and cytotoxic CD8+T cells and contribute to the phenotype of anti-inflammatory macrophages and the resolution of inflammation [53].

A reduction in Treg/Th17 cell ratios is consistently linked with chronic inflammation, the promotion of atherosclerosis, and vulnerable plaque syndromes. At later phases, an imbalanced ratio of immune cells results in an unresolved chronic inflammatory condition. This, in turn, leads to the formation of clinically unstable plaques characterized by large necrotic nuclei due to VSMCs and the death of macrophages, which are covered with thin fibrous caps [54].

## 4. Reverse Cholesterol Transport and Atherosclerosis

Due to a targeted decrease in apolipoprotein B-containing lipoproteins, the indicators of atherosclerotic cardiovascular diseases have been enhanced. Nevertheless, atherosclerotic cardiovascular diseases (ASCVDs) invariably remain the major cause of death. Moreover, randomly selected participants in modern lipid-lowering clinical trials also experience atherosclerotic cardiovascular disease events with critical frequency [55]. A while ago, attention began to focus on reverse cholesterol transfer; that is, the active cholesterol elimination cholesterol from the arterial wall. Reverse cholesterol transport is an endogenous mechanism by which cells export cholesterol and maintain homeostasis. Studies on both animals and humans give a clear idea that a violation of reverse cholesterol transport can result in faster development of atherosclerosis and, conversely, enhanced reverse cholesterol transport is able to limit or completely exclude the development of atherosclerosis [56].

The initial stage of cholesterol transfer from the cell to the acceptor, usually HDL particles, is the most studied aspect of reverse cholesterol transport. Experimental studies conducted on mice have shown that the macrophage-specific cholesterol efflux capacity (CEC) is accidentally associated with atherosclerosis. Ex vivo CEC measurement methods provided an opportunity to assess the ability of plasma or serum to take cholesterol from standardized cell lines as an indicator of CEC, a novel marker of HDL function that may be related to the risk of developing atherosclerotic cardiovascular diseases [57]. In fact, numerous observational studies among large cohorts of people have shown an inverse relationship between the baseline CEC level and CVD cases and even mortality from cardiovascular diseases in several cohorts with a high risk share. In order to determine whether this hand-targeting strategy can improve CV results, novel infusion agents that directly affect the CEC are being tested and in human trials. However, atherosclerosis develops unevenly throughout the vascular tree. In addition, arterial plaques differ in a range of morphological features, and depending on the stage of development, they are prone to rupture and have a tendency to thrombosis [58]. So how exactly does reverse cholesterol transport affect atherosclerosis in various vascular beds and play with architectural diversity? If the CEC and other reverse cholesterol transport indicators acquire clinical significance as predictors of ASCVD and therapy goals, these two issues become of great importance [28]. 

The study conducted by Shea and his colleagues in this issue of *ATVB* helps to fill in the missing knowledge using the observational nested design of the case-control study, which was applied in the Multi-Ethnic Study of Atherosclerosis (MESA), a large cohort of the middle-aged population who did not have cardiovascular disease at the baseline level [59]. The analysis, which included 465 coincident cases of cardiovascular disease, demonstrated stable feedback between baseline cholesterol emissions and cases of cardiovascular disease. When results were stratified by vascular territory, the inverse association was due solely to a 28% decrease in the probability of coronary events to an increase in the standard deviation in CEC while there was no clear association with stroke [60]. The sustained link with coronary events coincides with the results of earlier observational studies in low-risk populations in the Dallas Heart Study and EPIC-Norfolk and coincides with cross-sectional studies that reported inverse associations with the severity of advanced coronary atherosclerosis. Although this link between reverse cholesterol transport disorder and coronary atherosclerosis is quite obvious and quite consistent, only very recently has research put forward the suggestion that reverse cholesterol transport has an effect on the architecture of coronary plaques [61]. The load on the lipid plaques, calcification, and fibroadenoma of the thin membrane demonstrate the overall load on the plaques. The menace of plaque rupture and acute coronary syndrome is most reliably predicted by fibroateromas of the thin membrane and non-calcified plaques. For this reason, several cross-sectional studies conducted in people with an increased risk of CVD or undergoing clinically demonstrated coronary angiography have shown that CEC violation is linked with the prevalence of both fibroatheroma of the thin membrane and non-calcified plaque; at the same time, large and small cohorts failed to identify a connection between CEC and calcified coronary plaque [62].

This study offered a very interesting conclusion about the absence of a link with CVD. Firstly, the initial outflow was not associated with stroke. Secondly, it was also not essentially associated with the progression of carotid plaque, as was found with the help of an ultrasound in B-mode. Although cerebrovascular disease and CAD have many common risk factors, several key differences in the atherobiology of these vascular beds still exist [63]. The phenotype of cerebrovascular disease is more heterogeneous, including (1) cerebral infarction mediated by ischemic plaque, (2) cerebral infarction mediated by ischemic embolism, or (3) hemorrhagic cerebral infarction as a result of rupture of the arterial wall (not plaques) [64].

The processes of the last two results are distinctly different from the classical process mediated by plaques. However, clinical studies evaluating cerebrovascular diseases, for the most part, do not separate these disparate stroke events and decrease the ability to distinguish between factors directly related to the development of cerebrovascular plaques. In addition, in studies conducted among people, atherosclerosis of the carotid arteries is usually assessed by the thickness of the intima of the carotid artery or the presence of a carotid plaque using ultrasound or magnetic resonance imaging. In many large cohorts, the thickness of the intima of the carotid artery is actually a weak harbinger of stroke, and the presence of carotid plaque is a reliable harbinger of ischemic stroke events [65].

It is therefore not surprising that the results of human studies proving the link between cholesterol release and cerebrovascular diseases were heterogeneous. Earlier reports on a cross-section of samples revealed an inverse relationship between the widespread thickness of the intima of the carotid artery and the widespread stenosis of the carotid artery. Unlike these inverse associations in small and limited research projects, the current study in this issue of *ATVB* does not report a significant association with stroke or progression of carotid plaques in a large population cohort that has been observed longitudinally for many years [66].

It is very important that the researchers were able to analyze various subtypes of stroke (out of ~200 strokes, ~120 were not caused by hemorrhage or embolic events) and convincingly demonstrate no association with non-embolic ischemic stroke. In the Dallas Heart study (a younger population with fewer events took part), the ability to outflow was inversely related to stroke in secondary analyses [67]. Despite this, the number of stroke cases was small (N ~ 30) and was not analyzed by stroke subtype, and the outflow analyses used in this study had only minor differences compared to this study. Therefore, the current study at MESA is the largest and most epidemiologically rigorous to date regarding the assessment of the contribution of reverse cholesterol transport to the development of cerebrovascular plaques [68].

## 5. Conclusions

Thus, there is probably no important connection between reverse cholesterol transport, which has an effect on the ability of macrophages to remove cholesterol, and on cerebrovascular plaques and ischemic stroke. Nowadays, epidemiological studies show a stable relationship between impaired cholesterol outflow and coronary atherosclerosis, especially noncalcified plaques or thin-cap fibroatheroma. To understand the role of reverse cholesterol transport in atherosclerosis in other vascular pathologies, such as aorto-iliac disease, peripheral artery disease of the lower extremities, and graft vasculopathy, follow-up studies in large unselected cohorts, such as the current analysis in MESA, are required.

The search for treatment methods that can increase the level of HDL in the fields of lipoprotein metabolism and cardiovascular diseases has been ongoing for quite a long time. Previously, doctors often prescribed drugs to elevate the level of HDL-C. Currently, it is believed that these treatments are ineffective at reducing the risk of CVD. In several clinical studies, it has not been proven that elevating HDL cholesterol (for instance, with niacin or CETP suppression) enhances CVD outcomes, and randomized Mendelian studies also show that levels of HDL-C are not prognostic for cardiovascular disease events. These and other studies mean that, despite the presence of a large number of achievements in the development of various treatments that reduce LDL cholesterol, which have resulted in positive clinical results, there are no comparable achievements in strategies to increase the effectiveness of reverse cholesterol transport by elevating the HDL-C level. A case study of the need for such an enhancement, independent of the level of HDL-C, exists in the CETP inhibitor test data. Probably, this may be more than just a coincidence: the inability of torcetrapib to reduce the frequency of CVD events, despite an increase in HDL-C by ~72%, was also associated with its inability to stimulate whole-body reverse cholesterol transport when analyzing fecal sterol excretion.

It is noteworthy that in none of the above studies, nor in many similar studies, was the function of HDL established. Thus, there is a possibility that HDL function is a key attribute of reducing the risk of CVD. The function of HDR as a clinically essential factor has been supported not only in the aforementioned CEC studies, but also partially approved in some but not all studies of infusions of recombinant HDL and HDL-like particles. It is interesting that nowadays, all studies are of limited importance because they were not long enough to assess the impact on the outcomes of CVD, or were very small in the number of participants, or for both reasons. For instance, in one modest study (only 20 people participated), intravenous infusion of 1 dose of recovered HDL resulted in acute changes in plaques in the superficial femoral artery with a decrease in lipid content, macrophage size, and inflammation indicators. In another more extensive study of patients with acute coronary syndromes (ACS; 47 subjects completed the protocol), 5 weekly injections of a recombinant HDL-like particle (designated ETC-216) containing ApoA-I_milan_ led to a reduction in coronary atheroma volume by 4.2% compared to the baseline level measured by intravascular ultrasound.

An almost identical study was conducted with a formulation of wild-type ApoA-I (designated CSL111). The results were the same as in the ETC-216 study, in which the plaque volume was not significantly reduced (3.4%), which is possible because the course of treatment was quite short (four weeks) or because of other differences between the studies. Again, none of the studies provided data on the outcome of CVD. Thus, significant attention is being focused on the AEGIS II trial, in which apoA-I in a proprietary lipid formulation used to simulate HDL particles (CSL112) is administered to subjects with ACS (acute coronary syndrome). With an expected number of people (17,400), all participants will be randomly selected to receive either CSL112 or a placebo. The medicamentation/placebo will be administered once a week for four consecutive weeks by intravenous infusion. The first occurrence of either a major adverse cardiovascular event (MACE), or death from CD, or MI, or stroke within 90 days will be considered the primary endpoint. The expected completion date is 2022. 

Despite the discrepancies, it is believed that an elevation in the level of functional HDL in people at risk of developing CVD may still be a successful therapy to suppress the accelerated development of atherosclerosis and contribute to its regression. This opinion is based on the identified biological effects of functional HDL, which have been generalized, and on the results of clinical studies; however, they do not fully correspond to the final tests, in particular regarding the relationship between increased functional HDL and MACE levels. This causes a parallel need for additional studies, which the AEGIS II type represents, and for mechanical studies to further determine the factors that regulate the effect of HDL on cardiovascular disease, regardless of the concentration of HDL-C in plasma.

## Data Availability

Not applicable.
